# Long-term recovery following critical illness in an Australian cohort

**DOI:** 10.1186/s40560-018-0276-x

**Published:** 2018-02-05

**Authors:** Kimberley J. Haines, Sue Berney, Stephen Warrillow, Linda Denehy

**Affiliations:** 1 Physiotherapy Department, Western Health, Furlong Road, St. Albans, VIC 3021 Australia; 2 Department of Physiotherapy, Melbourne School of Health Sciences, The University of Melbourne, 200 Berkeley Street, Parkville, VIC 3010 Australia; 3 Department of Physiotherapy, Austin Hospital, 145 Studley Road, Heidelberg, VIC 3084 Australia; 4 Department of Intensive Care, Austin Hospital, 145 Studley Road, Heidelberg, VIC 3084 Australia

**Keywords:** Critical illness, Long-term outcomes

## Abstract

**Background:**

Almost all data on 5-year outcomes for critical care survivors come from North America and Europe. The aim of this study was to investigate long-term mortality, physical function, psychological outcomes and health-related quality of life in a mixed intensive care unit cohort in Australia.

**Methods:**

This longitudinal study evaluated 4- to 5-year outcomes. Physical function (six-minute walk test) and health-related quality of life (Short Form 36 Version 2) were compared to 1-year outcomes and population norms. New psychological data (Center for Epidemiological Studies–Depression, Impact of Events Scale) was collected at follow-up.

**Results:**

Of the 150 participants, 66 (44%) patients were deceased by follow-up. Fifty-six survivors were included with a mean (SD) age of 64 (14.2). Survivors’ mean (SD) six-minute walk distance increased between 1 and 4 to 5 years (465.8 m (148.9) vs. 507.5 m (118.2)) (mean difference = − 24.5 m, CI − 58.3, 9.2, *p* = 0.15). Depressive symptoms were low: median (IQR) score of 7.0 (1.0–15.0). The mean level of post-traumatic stress symptoms was low—median (IQR) score of 1.0 (0–11.0)—with only 9 (16%) above the threshold for potentially disordered symptoms. Short-Form 36 Physical and Mental Component Scores did not change between 1 and 4 to 5 years (46.4 (7.9) vs. 46.7 (8.1) and 48.8 (13) vs. 48.8 (11.1)) and were within a standard deviation of normal.

**Conclusions:**

Outcomes of critical illness are not uniform across nations. Mortality was increased in this cohort; however, survivors achieved a high level of recovery for physical function and health-related quality of life with low psychological morbidity at follow-up.

**Trial registration:**

The trial was registered with the Australian New Zealand Clinical Trials Registry ACTRN12605000776606.

**Electronic supplementary material:**

The online version of this article (10.1186/s40560-018-0276-x) contains supplementary material, which is available to authorized users.

## Background

Survivorship is the defining challenge of the twenty-first century in critical care [1] with increasing numbers of survivors experiencing new or worsened morbidity following critical illness [2, 3]. Attention has therefore focused on the quality of survivorship with adverse physical function, cognition and mental health outcomes, now recognised as post-intensive care syndrome (PICS) [1, 4, 5].

Existing long-term data suggests that ICU survivorship is associated with considerable long-term morbidity. Five-year data from North America indicate new and continued disability in physical function, cognition, and health-related quality of life (HRQoL) following adult respiratory distress syndrome (ARDS) [6, 7] and sepsis [2, 3, 6]. Five-year data from the United Kingdom (UK) and Europe indicate that in older, general ICU cohorts, survivors return to their pre-ICU HRQoL levels [8, 9], although often below population norms. Five-year data from other regions of the world are lacking. Previous findings may not be generalizable to other settings due to differences in models of care, patient cohorts, and differences in population HRQoL outcomes [10, 11].

Given that ageing of the population is a global phenomenon, there are calls to define critical care survivorship in a way similar to cancer and stroke survivorship [2]. Current data on recovery from critical illness is incomplete and predominantly limited to the Northern Hemisphere. Intensive care unit (ICU) follow-up studies are typically clustered around the short and medium term of 6 [10, 12–15] to 12 months [14, 16–18] and typically describe HRQoL and physical function outcomes. Little is known about the overall experience for patients at 5 years, with a particular paucity of data relating to psychological outcomes beyond 1 to 2 years [13, 19–22].

Within Australia, no published long-term outcome data extends beyond 1 year [14, 17]. Comprehensive survivorship data from other healthcare contexts is important to contribute to the global understanding of critical care survivorship and improve generalizability across settings. Therefore, the primary aim of this study was to:

i) Investigate long-term mortality, physical function, psychological outcomes, and HRQoL in a mixed ICU cohort in Australia

The secondary aims of this study were to:

i) Compare the long-term physical function of Australian survivors with 1-year post-ICU physical function
ii) Investigate the long-term prevalence of symptoms of anxiety, depression, and post-traumatic stress disorder (PTSD) in Australian ICU survivors
iii) Compare the long-term HRQoL of Australian survivors with pre- and 1 year post-ICU
iv) Investigate long-term return to work and independent living status in Australian survivors

## Methods

### Study design, setting, and participants

This study was a prospective, observational follow-up study of a longer-stay (median ICU admission of 7 days) cohort. This randomised controlled trial (RCT) was conducted in a quaternary ICU in Melbourne, Australia, from 2007 to 2010 and detailed elsewhere [17]. RCT participants (*n* = 150) were screened and invited to participate, and informed consent sought for follow-up. Participants were included in the original RCT if they were > 18 years, ICU length of stay ≥ 5 days, understood English, resided within 50 km from the hospital and the intensive care specialist agreed to their participation. Patients were excluded if they had major disorders affecting the central nervous system or other conditions that would prevent participation in exercise, were approaching imminent death, length of stay > 5 days due to lack of general ward bed availability and unable to perform study physical outcome measures pre-morbidly.

The institutional ethics committee of Austin Health approved the study (H2012/04606), which is reported according to STROBE guidelines [23].

### Procedure

From May 2012–December 2013, all patients enrolled in the RCT were screened for survival using hospital and general practitioner records. Patients not confirmed deceased were sent a letter describing the study and inviting participation, with an opt-out clause. Patients were contacted a week later via telephone to seek consent.

Outcome measures were performed at 4 to 5 years following ICU discharge in a standardised hospital environment and questionnaires completed in-person by a single assessor (KH). If participants were not within travelling distance (> 1 h via car journey) of the hospital, questionnaires were completed by phone interview. If participants were within travelling distance (< 1 h via car journey), but could not travel, the outcome assessor attended their home.

### Outcome measurement

#### Demographic and 1-year follow-up data

Baseline demographic and 1-year follow-up data were drawn from the RCT [17] (Table 1). Additional demographic data was sought for the current study, including independent living status and employment status (devised questionnaire Additional file 1: Appendix E1), as well as the need for informal caregiver assistance following hospital discharge. ICU-acquired weakness (ICU-AW) was diagnosed when the Medical Research Council (MRC) score was less than 48/60 [24] and then dichotomized as either present or absent.

**Table 1:** Demographics

	Whole cohort (*n* = 150)	Survivor cohort (*n* = 56)	Deceased (*n* = 66)
Age (years) at recruitment mean (SD)	61 (15.8)	59 (14.1) 64 (14.2) at 4–5 years	67 (14.6)
Male, *n* (%)	94 (62)	34 (61)	44 (66)
APACHE II mean (SD)	20 (7)	18 (6)	22 (7.9)
ICU diagnosis (%)			
Pneumonia	17	13	21
Cardiac^a^	39	43	33
Other surgery	15	16	14
Liver disease/transplant	10	13	9
Sepsis	8	5	12
Other	8	10	11
> 1 comorbidity, *n* (%)	53 (35%)	17 (30%)	30 (46%)
MV hours median (IQR)	92 (26–165)	96 (0–689.3)	84 (41–186)
ICU length of stay (days) median (IQR)	7 (6–11)	7 (5–11)	8 (6–11)
Hospital length of stay (days) median (IQR)	22 (15–36)	19 (11.3–29.5)	25 (18–45)

^a^Includes cardiogenic shock, cardiac arrest and complicated cardiac surgery

#### Mortality

Mortality data was sourced from hospital databases where available and all mortality data cross-referenced with the state-based Victorian Births and Deaths Registry (completed June 24, 2014).

#### Performance-based tests and patient-reported outcomes

Physical function was measured using the six-minute walk test (6MWT), a standardised walking test to measure functional exercise capacity, previously used in ICU cohorts [6, 14, 17, 25] and the Timed Up and Go Test (TUG) to assess functional mobility [26]. Bilateral handgrip strength was assessed using hand held dynamometry, modelled on a previously published protocol for ICU patients [27].

Psychological outcomes were assessed in the following domains: depression, anxiety, and post-traumatic stress disorder (PTSD). Depression and anxiety symptoms were screened using the Hospital Anxiety and Depression Scale (HADS) [28], one of the most commonly used measures in the critically ill [29]. Subscale scores of 0–7 are normal and > 8 indicates clinically significant symptoms [28]. The Centre for Epidemiological Studies-Depression Scale (CES-D) [30] was also included as it is the only measure of depression validated against clinician diagnoses in the post-ICU setting [29]. A cut-off score of 16 or more was used to define clinically significant depression [30]. PTSD was assessed using the Impact of Event Scale (IES) [31], a 15-item questionnaire, and a cut-off score of 19 was reported against, as originally described [32] and consistent with previous studies [33].

HRQoL was measured using the Short Form-36 Questionnaire, version 2 (SF-36v2) [34], and the Assessment of Quality of Life (AQoL) questionnaire [35]. The SF-36v2 has been widely used and validated in the critically ill [36, 37] and consists of eight subscales (including physical functioning, bodily pain, social functioning and mental health). Two summary scores (physical and mental, PCS and MCS respectively) based upon population norms [37] are produced and presented as standardised *T*-scores (mean = 50 and standard deviation = 10) [38]. The AQoL is a 15-item generic health, multi-attribute utility instrument [35] which has been previously used [17] and validated in the critically ill [39]. The AQoL utility instrument boundaries range from − 0.04 (state worse than death) to 1.00 (full HRQoL).

### Statistical analysis

Descriptive data are presented as median [interquartile range (IQR)] and mean [standard deviation (SD)] as appropriate. Imputation of missing data for survivors was not undertaken as there was little missing data between baseline, 1-year, and 4–5-year time points for the outcomes of interest.

Multiple variable logistic regression was conducted as a post hoc analysis to investigate the factors associated with mortality at follow-up. A priori selected baseline variables were compared between survivors and non-survivors, using univariate analyses with *p* ≤ 0.1 used to determine which variables were entered into the final model. Six independent variables (baseline age, APACHE II scores, acute hospital length of stay, pre-ICU HRQoL (AQoL utility score), Physical Component Summary Score of the SF36v2, ability to perform the Physical Function in Intensive Care Test by day 10 of ICU admission [40]) were included in the final model. The Hosmer-Lemeshow test was used to assess goodness of fit, and pseudo *R*^2^ statistics were calculated with Nagelkerke *R*-square. The Wald test was used to assess the significance of the association of the individual variables with mortality. Odds ratios (95% CIs) and sensitivity and specificity of the model are reported. A Kaplan-Meier analysis was conducted to investigate differences in survival between those without comorbidities and those with one or more.

For repeated measures at one with either 4 or 5 years (e.g. 6MWT, TUG), the paired *t* test was used for normally distributed data and Wilcoxon signed-rank test for non-normally distributed data. One-way repeated measures ANOVA was used to compare HRQoL (AQoL utility score, SF36v2 PCS, MCS and PF) across the three time points. Where appropriate, analyses are reported as mean change in scores with 95% CI and compared with reported minimal clinically important differences as a secondary analysis, where available.

Data analysis was performed using SPSS™ (Mac SPSS™ Statistical Version 20, IBM, New York, NY) and *p* < 0.05 was taken to indicate statistical significance.

## Results

**Fig. 1:**
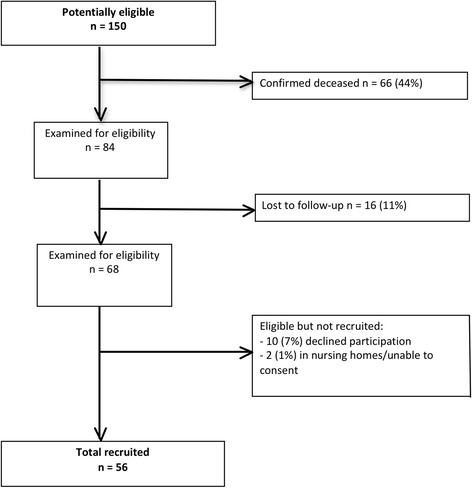
Patient follow-up status at 4–5 years post-ICU

Of the 150 patients in the original RCT, 84 were assessed for eligibility with 16 lost to follow-up. Of the 68 still alive at follow-up and able to be contacted, 56 (82% of 68) agreed to participate. At follow-up, the survivor cohorts had a mean (SD) age of 64 (14.2), were mostly male, were previously a moderately unwell cohort with mean (SD) APACHE II scores of 18 (6), and had been mechanically ventilated for a median of 4 days (Table 1). The flow of participants through the study is provided in Fig. 1.

### Mortality

**Fig. 2:**
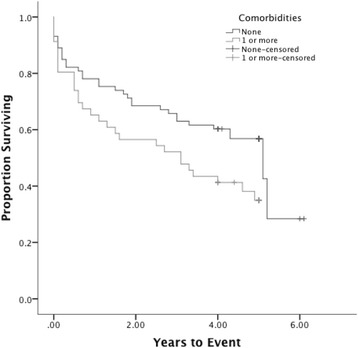
Kaplan-Meier curve for survival from 0 to 5 years for different numbers of premorbid comorbidities

In the entire group of 150, 66 (44%) patients were deceased (cause of death listed in Additional file 1: Appendix E2). Date of death was only available for 43 (65%) patients, and mortality was highest (*n* = 19, 44%) during the first year following ICU discharge. In the multivariable regression, those who were deceased by follow-up were older (*p* = 0.05), with higher APACHE II scores (*p* = 0.001) and comorbidities (46% with one or more) compared to survivors (Table 2). Survival rates were significantly improved in the group who had no comorbidities compared to those who had one or more (Fig. 2, log rank *p* = 0.03).

**Table 2:** Logistic regression predicting the likelihood of death by longer-term follow-up

	*B*	SE	Wald	df	*P*	OR	95% CI
Baseline MRC score	− 0.76	0.05	2.16	1	0.14	0.93	0.84 to 1.03
Baseline age	0.05	0.02	4.23	1	0.04*	1.05	1.00 to 1.10
APACHE II	0.17	0.07	6.46	1	0.01*	1.18	1.04 to 1.34
Acute hospital length of stay	0.12	0.01	1.59	1	0.21	1.01	0.99 to 1.03
Baseline AQoL utility score	− 2.16	1.28	2.85	1	0.09	0.12	0.01 to 1.41
Baseline SF36v2 PCS	− 0.04	0.03	1.58	1	0.21	0.96	0.91 to 1.02
Constant	0.41	3.47	0.01	1	0.91	1.51	

*B* beta coefficient, *SE* standard error, *Wald* Wald test, *df* degrees of freedom, *OR* odds ratio, *CI* confidence interval *MRC score* Medical Research Council score, *APACHE II* Acute Physiological and Chronic Health Evaluation II, *AQoL* Assessment of Quality of Life, *SF36v2 PCS* Short Form 36 Health Survey Version 2 Physical Component Score

*Statistically significant *p ≤* 0.05

### Physical function

At longer-term follow-up, 48 of the 56 survivors (86%) completed the 6MWT with data unable to be collected on 8 survivors due to the travel distance outside defined inclusion criteria. Whilst there was an improvement in the survivors’ 6MWT distance between 1 year (mean 465.8 m, SD 148.9) and 4–5 years (mean 507.5 m, SD 118.2), this difference was not statistically significant *p* = 0.15 (mean difference = − 24.5 m, CI − 58.3, 9.2). Survivors’ 6MWT distance at 4–5 years was 70% of the predicted distance for Australian age and gender-matched norms [41]. In comparison, survivors’ scores were 89% of predicted North American normative values [42], derived from a sample size more than double that of the Australian reference equation.

**Fig. 3:**
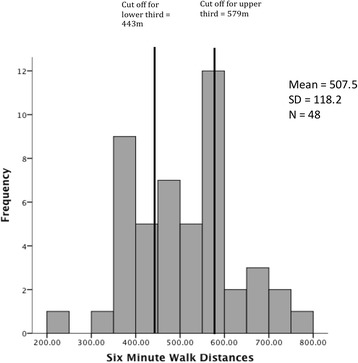
Histogram of frequency distribution for Six Minute Walk Distances

More than a third of survivors had an improvement in their walk distance greater than the reported minimal clinically important difference (MCID) of 20 m as reported for ICU survivors [43] and similarly for the previously reported MCID of 30 m for patients with chronic respiratory disease [44, 45]. The frequency distribution of distances for the 6MWT is displayed in Fig. 3.

The survivors had an improvement in their TUG time from a median (IQR) of 7.5 s (6.0–9.0) at 1 year to 6.5 s (6.0–9.0) at 4–5 years. This improvement was statistically significant (*p* = 0.001) and survivors outperformed normative values for their age range (mean (CI) 8.1 (7.1–9.0) [46] although did not improve by one of the few available reports of MCID for the TUG in acutely hospitalised older medical patients of 9.5 s [47].

Baseline outcome measures for strength included Medical Research Council (MRC) scores and diagnosis of ICU-AW. Survivors’ mean (SD) MRC score was 51/60 (8.0), and 73% did not have ICU-AW as measured during their ICU admission. At longer-term follow-up, grip strength in males was 76% of age-matched normative values [48] at mean (SD) 34 (12.5) kg. Females had a mean (SD) grip strength of 20 (9.9) kg, which was 77% of age-matched normative values [48].

### Psychological outcomes

As measured by the CES-D, depressive symptoms were low with a median (IQR) CES-D score of 7.0 (1.0–15.0). Forty (71%) survivors had no depression, 10 (18%) had mild depression and 6 (11%) had major depression. As measured by the HADS, survivors’ symptoms of anxiety and depression were within normal ranges with respective median (IQR) scores of 3.0 (1.0–6.0) and 1.0 (0–4). Forty-five (80%) survivors had no symptoms of anxiety, whilst 11 (20%) had clinically significant symptoms. Forty-eight (86%) survivors reported no symptoms of depression whilst 8 (14%) had clinically significant symptoms. The incidence of PTSD was also sub-clinical with a median (IQR) score of 1.0 (0–11.0) as measured by the IES. Nine (16%) survivors had ‘clinically significant’ symptoms for PTSD.

### Health-related quality of life

At follow-up, survivors’ mean SF36v2 PCS scores were normal for age-matched Australian values (Table 3), whilst the MCS were below population normative values but within one SD [38]. For survivors with available data at both time points, there was no significant difference in PCS (*p* = 0.32, *n* = 37) over time although there was a significant improvement for the MCS (*p* = 0.01, *n* = 37). Only the differences in MCS between pre-ICU and 1 year follow-up exceeded the reported MCID of 5-point difference [37].

**Table 3:** Descriptive statistics for health-related quality of life scores over time for survivors and age-matched normative values

Outcome measure	Baseline		1 year		4–5 years		Age-matched Australian normative values^a^
	*n*	Mean (SD)	*n*	Mean (SD)	*n*	Mean (SD)	Mean (SD)
AQol utility	43	0.70 (0.25)	49	0.77 (0.24)	56	0.74 (0.23)	0.79 (0.19)
SF36v2 PCS	43	43.4 (12.1)	44	46.4 (7.9)	56	46.7 (8.1)	46.8 (11.6)
SF36v2 MCS	43	42.9 (12.8)	44	48.8 (13)	56	48.8 (11.1)	50.1 (10.8)
Physical functioning	43	45.3 (12.3)	44	44.7 (10)	56	43.6 (11.5)	47.4 (10.7)
Role physical	43	40.4 (14.1)	44	46.0 (10.1)	56	46.3 (11.6)	47.5 (12.0)
Bodily pain	43	47.2 (15.7)	44	53.4 (11.0)	56	50.7 (10.7)	47.3 (10.4)
General health	43	41.6 (10.5)	44	44.2 (9.9)	56	45.6 (9.9)	47.4 (11.9)
Vitality	43	39.7 (13.3)	44	46.6 (11.9)	56	50.7 (8.4)	49.0 (10.9)
Social functioning	43	42.3 (15.1)	44	48.4 (12.9)	56	50.0 (8.8)	49.3 (11.1)
Role emotional	43	45.4 (15.3)	44	48.5 (11.5)	56	44.2 (14.3)	49.2 (11.5)
Mental health	43	42.2 (13.5)	44	48.5 (14.2)	56	49.2 (12.0)	49.4 (11.2)

*AQoL utility* Assessment of Quality of Life Utility score range − 0.04 (state worse than death) to 1.00 (perfect health), *SF36v2* Short Form 36 Health Survey version 2 in which higher scores indicate greater performance and data is presented as *T* scores where the population mean is 50 and the SD is 10, *PCS* Physical Component Score, *MCS* Mental Component Score, *PF* Physical Function Subscale

^a^Age-matched Australian population for mean (SD) age 64 (14.2) of survivors at 4–5-year follow-up

At longer-term follow-up survivors’ mean (SD), AQoL utility score was 0.74 (0.23), below age-matched normative values of 0.79 (0.19) [49]. There were no differences in AQoL scores over time (*p* = 0.14, *n* = 38). Between pre-ICU and 1-year follow-up, the change in the survivors’ mean difference in AQoL scores exceeded the reported MCID of 0.06 [49]. Between 1 year and 4–5 years, there was a smaller clinically insignificant improvement. Between 1 and 4 or 5-year follow-up, 11 (23%) survivors improved more than the AQoL MCID [49], 22 (45%) deteriorated and 16 (32%) did not differ compared to the MCID.

### Return to work

Twenty (69%) survivors who had been working prior to ICU (*n* = 29, 52% of original cohort) had returned to work. Five (17%) survivors had not, reporting poor health as the reason. Twenty-seven (48%) survivors were not working prior to ICU, with 21 (81%) being retired whilst only 4 (15%) survivors were not working due to ill health.

## Discussion

This first longitudinal Australian study provides a contrasting perspective to other international reports of critical care survivorship [2, 3, 6–8, 50]. Survivors were characterised by a low burden of impairments in their physical, HRQoL and psychological outcomes. This study comprehensively evaluated multiple outcomes including physical function and HRQoL in a long-stay, heterogeneous cohort representative of Australian ICUs [51]. It is also one of the first reports to provide empirical psychological data at 4 to 5 years. A particular strength is the combined use of performance-based and patient-reported measurement, an important consideration in ICU follow-up studies [52].

### Mortality

The long-term mortality rate of 44% was unexpectedly high compared to previous reports of 19% [6] and 30% [8] in landmark studies at 5-year follow-up. Differences observed in our study may be attributable to increased baseline age and higher APACHE II scores comparative to these previous reports [6, 8]. The Kaplan-Meier curve highlights the contribution of comorbidity to mortality although these analyses were not adjusted for age and APACHE II scores. Overall, the original cohort had a high prevalence of comorbid illness [53]. Almost half of the non-survivors had one or more comorbidities compared to a third of survivors. Pre-existing comorbid illness may be an important consideration for post-ICU trajectories of recovery [53] with worse outcomes attributed to pre-existing illness, particularly for HRQoL [54, 55]. We hypothesise our observed mortality rate could be influenced by local healthcare system factors including physician decision-making regarding ICU admission and rationing and socioeconomic factors. For example, the Australian healthcare model may be relatively well resourced compared to other regions, with a greater ratio of ICU beds to ward beds, a ‘closed’ ICU model and 1:1 nurse to patient ratios [11]. As a result, the threshold for ICU admission may be lower in Australian units than more resource-limited regions.

### Physical function

The majority of survivors had minimal decrements in their physical function as evidenced by their 6MWD and TUG values relative to population normative data. Most recovery appears to be gained by 1 year although this was a heterogeneous group, with some survivors still experiencing physical impairment at follow-up. This demonstrates the variability in trajectories of recovery [56] that may influence response to targeted intervention [57, 58] and the importance of stratification according to disability [59]. The greatest deficits were seen in the survivors’ grip strength compared to normative data although this is difficult to interpret as the majority did not have an earlier diagnosis of ICU-AW and grip strength was not measured during the original RCT. Reasons for observed differences in our physical function outcomes could be related to a high proportion of patients admitted for cardiac causes. These patients have a relatively unimpeded recovery following surgery and critical care with resolution of some premorbid comorbidities [10, 60]. The majority of our cohort did not have a diagnosis of ICU-AW during their ICU admission, and this may explain the overall level of high performance in the physical function tests. Comparatively, Herridge and colleagues hypothesised that in their younger cohort with a lower prevalence of comorbid illness, the adverse physical outcomes observed in their cohort likely stemmed from persistent weakness although the incidence of ICU-AW was not specifically reported [6].

### HRQoL

The HRQoL findings of our study were comparable to the patterns demonstrated in the study by Cuthbertson and colleagues [8]. In both of these studies, by 5 year follow-up, survivors’ HRQoL scores were comparable if not better than their premorbid scores. However, in the study by Cuthberston and colleagues, the survivors’ HRQoL remained lower than population norms at 5 years whereas in our study, scores were comparable to Australian population norms at this time point which is consistent with the findings of more recent research [9, 61]. The similarity in trends in the SF36 scores over time between these two studies is likely attributable to similarities in demographics (both were conducted in mixed older age cohorts with comparable APACHE II scores) and consistency in the administration of the SF36 to capture premorbid HRQoL.

### Psychological outcomes

We provide one of the first reports at 4 to 5 years of directly measured, comprehensive psychological outcome data. Consistent with the other outcomes we have described for this cohort, the incidence and prevalence of psychological morbidity was low. Although not evaluated, these survivors may have possessed higher levels of attributes such as resiliency and self-efficacy as well as access to greater familial and social support. This may have assisted their overall high level of recovery. This concept has been demonstrated in other ICU survivors where resilience has an inverse correlation with neuropsychological impairment and other outcomes such as pain and self-care [62].

Our data are influenced by survivor bias and loss to follow-up inherent in longitudinal studies. The findings suggest most recovery occurs within the first year, and this may be important to consider in the design of future interventional studies. Patients and their families may be at greatest risk of adverse outcomes during this time, and promising interventions such as peer support may assist their recovery transition [63]. A limitation of this study is the lack of follow-up from 1 to 4 and 5 years. This may have assisted in improved follow-up rates through repeated contact with participants although overall attrition was comparable if not better than previous studies. We approached the measurement of psychological outcomes using screening tools rather than diagnostic tools although this was consistent with other reports [21, 22, 29, 33, 64]. Further, the IES was selected as the best measure to screen for PTSD at the time of study design, although more recent reports support the use of the IES-Revised [65] which may limit comparability of our findings.

This study highlights the need for a co-ordinated and collaborative international approach to describe the spectrum of critical care survivorship, particularly as we are starting to see better outcomes reported in other regions such as Europe [9]. In order to improve the outcomes for critical care survivors, consensus is required between clinicians, researchers and policy-makers regarding time points for follow-up and which outcome measures to use. Further, there may be other important factors to evaluate that mediate recovery such resilience [62] and post-traumatic growth and the role of caregivers and their ability to provide support following exposure to critical illness [66, 67]. By establishing large international datasets for a range of patient and family outcomes, we may be able to better understand survivorship from critical illness and develop interventions that will be sensitive to these specific domains.

## Conclusions

In conclusion, this Australian cohort had an increased mortality rate compared to existing studies that may be attributable to differences in healthcare models and delivery of care. However, survivors achieved recovery in their physical function and HRQoL comparable with population norms and had low psychological morbidity. Further exploration through large datasets is warranted to understand regional differences in outcomes to truly define critical care survivorship from an international perspective.

## Additional file

Additional file 1: Appendix E1. Independent living status and employment status questions. Appendix E2. Cause of death information provided by the Victorian Births and Deaths Registry. (DOC 44 kb)

## Abbreviations

6MWT: Six-minute walk test
AQoL: Assessment of Quality of Life
ARDS: Adult respiratory distress syndrome
CES-D: Centre for Epidemiological Studies–Depression Scale
HADS: Hospital Anxiety and Depression Scale
HR-QoL: Health-related Quality of Life
ICU: Intensive care unit
ICU-AW: Intensive care unit-acquired weakness
IES: Impact of events scale
MCID: Minimal clinically important difference
MCS: Mental Component Summary Scale (of SF-36)
MRC: Medical Research Council
PCS: Physical Component Summary Scale (of SF-36)
PICS: Post-intensive care syndrome
PTSD: Post-traumatic stress disorder
RCT: Randomised controlled trial
SF-36: Short Form-36
STROBE: STrengthening the Reporting of OBservational Studies in Epidemiology
TUG: Timed Up and Go Test
UK: United Kingdom

## References

[1] Iwashyna TJ (2010). Survivorship will be the defining challenge of critical care in the 21st century. Ann Intern Med.

[2] Iwashyna TJ, Cooke CR, Wunsch H, Kahn JM (2012). Population burden of long-term survivorship after severe sepsis in older Americans. J Am Geriatr Soc.

[3] Iwashyna TJ, Ely EW, Smith DM, Langa KM (2010). Long-term cognitive impairment and functional disability among survivors of severe sepsis. JAMA.

[4] Elliott D, Davidson JE, Harvey MA, Bemis-Dougherty A, Hopkins RO, Iwashyna TJ, Wagner J, Weinert C, Wunsch H, Bienvenu OJ, Black G, Brady S, Brodsky MB, Deutschman C, Doepp D, Flatley C, Fosnight S, Gittler M, Gomez BT, Hyzy R, Louis D, Mandel R, Maxwell C, Muldoon SR, Perme CS, Reilly C, Robinson MR, Rubin E, Schmidt DM, Schuller J, Scruth E, Siegal E, Spill GR, Sprenger S, Straumanis JP, Sutton P, Swoboda SM, Twaddle ML, Needham DM (2014). Exploring the scope of post-intensive care syndrome therapy and care: engagement of non-critical care providers and survivors in a second stakeholders meeting. Crit Care Med.

[5] Needham DM, Davidson J, Cohen H, Hopkins RO, Weinert C, Wunsch H, Zawistowski C, Bemis-Dougherty A, Berney SC, Bienvenu OJ, Brady SL, Brodsky MB, Denehy L, Elliott D, Flatley C, Harabin AL, Jones C, Louis D, Meltzer W, Muldoon SR, Palmer JB, Perme C, Robinson M, Schmidt DM, Scruth E, Spill GR, Storey CP, Render M, Votto J, Harvey MA (2012). Improving long-term outcomes after discharge from intensive care unit: report from a stakeholders' conference. Crit Care Med.

[6] Herridge MS, Tansey CM, Matte A, Tomlinson G, Diaz-Granados N, Cooper A, Guest CB, Mazer CD, Mehta S, Stewart TE, Kudlow P, Cook D, Slutsky AS, Cheung AM, Canadian Critical Care Trials G (2011). Functional disability 5 years after acute respiratory distress syndrome. N Engl J Med.

[7] Pfoh ER, Wozniak AW, Colantuoni E, Dinglas VD, Mendez-Tellez PA, Shanholtz C, Ciesla ND, Pronovost PJ, Needham DM (2016). Physical declines occurring after hospital discharge in ARDS survivors: a 5-year longitudinal study. Intensive Care Med.

[8] Cuthbertson BH, Roughton S, Jenkinson D, Maclennan G, Vale L (2010). Quality of life in the five years after intensive care: a cohort study. Crit Care.

[9] Hofhuis JG, van Stel HF, Schrijvers AJ, Rommes JH, Spronk PE (2015). ICU survivors show no decline in health-related quality of life after 5 years. Intensive Care Med.

[10] Skinner EH, Warrillow S, Denehy L (2011). Health-related quality of life in Australian survivors of critical illness. Crit Care Med.

[11] Bellomo R, Stow PJ, Hart GK (2007). Why is there such a difference in outcome between Australian intensive care units and others?. Curr Opin Anaesthesiol.

[12] Garland A, Dawson NV, Altmann I, Thomas CL, Phillips RS, Tsevat J, Desbiens NA, Bellamy PE, Knaus WA, Connors AF, Investigators S (2004). Outcomes up to 5 years after severe, acute respiratory failure. Chest.

[13] Jackson JC, Pandharipande PP, Girard TD, Brummel NE, Thompson JL, Hughes CG, Pun BT, Vasilevskis EE, Morandi A, Shintani AK, Hopkins RO, Bernard GR, Dittus RS, Ely EW, Bringing to light the Risk F, Incidence of Neuropsychological dysfunction in ICUssi (2014). Depression, post-traumatic stress disorder, and functional disability in survivors of critical illness in the BRAIN-ICU study: a longitudinal cohort study. Lancet Respir Med 2:369–79 doi: 10.1016/S2213-2600(14)70051-7. 10.1016/S2213-2600(14)70051-7 PMC4107313 24815803

[14] Elliott D, McKinley S, Alison J, Aitken LM, King M, Leslie GD, Kenny P, Taylor P, Foley R, Burmeister E (2011). Health-related quality of life and physical recovery after a critical illness: a multi-centre randomised controlled trial of a home-based physical rehabilitation program. Crit Care.

[15] Investigators TS, Hodgson C, Bellomo R, Berney S, Bailey M, Buhr H, Denehy L, Harrold M, Higgins A, Presneill J, Saxena M, Skinner E, Young P, Webb S (2015). Early mobilization and recovery in mechanically ventilated patients in the ICU: a bi-national, multi-centre, prospective cohort study. Crit Care.

[16] Cuthbertson BH, Scott J, Strachan M, Kilonzo M, Vale L (2005). Quality of life before and after intensive care. Anaesthesia.

[17] Denehy L, Skinner EH, Edbrooke L, Haines K, Warrillow S, Hawthorne G, Gough K, Hoorn SV, Morris ME, Berney S (2013). Exercise rehabilitation for patients with critical illness: a randomized controlled trial with 12 months of follow-up. Crit Care.

[18] Herridge MS, Cheung AM, Tansey CM, Matte-Martyn A, Diaz-Granados N, Al-Saidi F, Cooper AB, Guest CB, Mazer CD, Mehta S, Stewart TE, Barr A, Cook D, Slutsky AS, Canadian Critical Care Trials G (2003). One-year outcomes in survivors of the acute respiratory distress syndrome. N Engl J Med.

[19] Duggan MC, Wang L, Wilson JE, Dittus RS, Ely EW, Jackson JC (2017). The relationship between executive dysfunction, depression, and mental health-related quality of life in survivors of critical illness: results from the BRAIN-ICU investigation. J Crit Care.

[20] Jackson JC, Girard TD, Gordon SM, Thompson JL, Shintani AK, Thomason JW, Pun BT, Canonico AE, Dunn JG, Bernard GR, Dittus RS, Ely EW (2010). Long-term cognitive and psychological outcomes in the awakening and breathing controlled trial. Am J Respir Crit Care Med.

[21] Needham DM, Dinglas VD, Bienvenu OJ, Colantuoni E, Wozniak AW, Rice TW, Hopkins RO, Network NNA (2013). One year outcomes in patients with acute lung injury randomised to initial trophic or full enteral feeding: prospective follow-up of EDEN randomised trial. BMJ.

[22] Nikayin S, Rabiee A, Hashem MD, Huang M, Bienvenu OJ, Turnbull AE, Needham DM (2016). Anxiety symptoms in survivors of critical illness: a systematic review and meta-analysis. Gen Hosp Psychiatry.

[23] von Elm E, Altman DG, Egger M, Pocock SJ, Gotzsche PC, Vandenbroucke JP, Initiative S (2008). The Strengthening the Reporting of Observational Studies in Epidemiology (STROBE) statement: guidelines for reporting observational studies. J Clin Epidemiol.

[24] De Jonghe B, Sharshar T, Lefaucheur JP, Authier FJ, Durand-Zaleski I, Boussarsar M, Cerf C, Renaud E, Mesrati F, Carlet J, Raphael JC, Outin H, Bastuji-Garin S, Groupe de Reflexion et d'Etude des Neuromyopathies en R (2002). Paresis acquired in the intensive care unit: a prospective multicenter study. JAMA.

[25] Holland AE, Spruit MA, Troosters T, Puhan MA, Pepin V, Saey D, McCormack MC, Carlin BW, Sciurba FC, Pitta F, Wanger J, MacIntyre N, Kaminsky DA, Culver BH, Revill SM, Hernandes NA, Andrianopoulos V, Camillo CA, Mitchell KE, Lee AL, Hill CJ, Singh SJ (2014). An official European Respiratory Society/American Thoracic Society technical standard: field walking tests in chronic respiratory disease. Eur Respir J.

[26] Podsiadlo D, Richardson S (1991). The timed “up & go”: a test of basic functional mobility for frail elderly persons. J Am Geriatr Soc.

[27] Baldwin CE, Paratz JD, Bersten AD (2013). Muscle strength assessment in critically ill patients with handheld dynamometry: an investigation of reliability, minimal detectable change, and time to peak force generation. J Crit Care.

[28] Zigmond AS, Snaith RP (1983). The hospital anxiety and depression scale. Acta Psychiatr Scand.

[29] Davydow DS, Gifford JM, Desai SV, Bienvenu OJ, Needham DM (2009). Depression in general intensive care unit survivors: a systematic review. Intensive Care Med.

[30] Radloff LS (1977). The CES-D scale: a self-report depression scale for research in the general population. Appl Psychol Meas.

[31] Griffiths J, Fortune G, Barber V, Young JD (2007). The prevalence of post traumatic stress disorder in survivors of ICU treatment: a systematic review. Intensive Care Med.

[32] Horowitz M, Wilner N, Alvarez W (1979). Impact of event scale: a measure of subjective stress. Psychosom Med.

[33] Davydow DS, Gifford JM, Desai SV, Needham DM, Bienvenu OJ (2008). Posttraumatic stress disorder in general intensive care unit survivors: a systematic review. Gen Hosp Psychiatry.

[34] Ware JEK MA, Dewey JE (2000). How to score version 2 of the SF-36 health survey.

[35] Hawthorne G, Richardson J, Osborne R (1999). The assessment of quality of life (AQoL) instrument: a psychometric measure of health-related quality of life. Qual Life Res.

[36] Chrispin PS, Scotton H, Rogers J, Lloyd D, Ridley SA (1997). Short form 36 in the intensive care unit: assessment of acceptability, reliability and validity of the questionnaire. Anaesthesia.

[37] Ware JE, Snow KK, Kosinski M, Gandek B (1993). SF-36 health survey: manual and interpretation guide.

[38] Hawthorne G, Osborne RH, Taylor A, Sansoni J (2007). The SF36 version 2: critical analyses of population weights, scoring algorithms and population norms. Qual Life Res.

[39] Skinner EH, Denehy L, Warrillow S, Hawthorne G (2013). Comparison of the measurement properties of the AQoL and SF-6D in critical illness. Crit Care Resusc.

[40] Skinner EH, Berney S, Warrillow S, Denehy L (2009). Development of a physical function outcome measure (PFIT) and a pilot exercise training protocol for use in intensive care. Crit Care Resusc.

[41] Jenkins S, Cecins N, Camarri B, Williams C, Thompson P, Eastwood P (2009). Regression equations to predict 6-minute walk distance in middle-aged and elderly adults. Physiother Theory Pract.

[42] Enright PL, Sherrill DL (1998). Reference equations for the six-minute walk in healthy adults. Am J Respir Crit Care Med.

[43] Chan KS, Pfoh ER, Denehy L, Elliott D, Holland AE, Dinglas VD, Needham DM (2015). Construct validity and minimal important difference of 6-minute walk distance in survivors of acute respiratory failure. Chest.

[44] Puhan MA, Gimeno-Santos E, Scharplatz M, Troosters T, Walters EH, Steurer J. Pulmonary rehabilitation following exacerbations of chronic obstructive pulmonary disease. Cochrane Database Syst Rev:CD005305. 2011; doi: 10.1002/14651858.CD005305.pub3. 10.1002/14651858.CD005305.pub3 21975749

[45] Holland AE, Nici L (2013). The return of the minimum clinically important difference for 6-minute-walk distance in chronic obstructive pulmonary disease. Am J Respir Crit Care Med.

[46] Bohannon RW (2006). Reference values for the timed up and go test: a descriptive meta-analysis. J Geriatr Phys Ther.

[47] de Morton NA, Keating JL, Jeffs K (2007) Exercise for acutely hospitalised older medical patients. Cochrane Database Syst Rev:CD005955 doi: 10.1002/14651858.CD005955.pub2. 10.1002/14651858.CD005955.pub3 PMC9648425 36355032

[48] Gunther CM, Burger A, Rickert M, Crispin A, Schulz CU (2008). Grip strength in healthy Caucasian adults: reference values. J Hand Surg Am.

[49] Hawthorne G, Osborne R (2005). Population norms and meaningful differences for the assessment of quality of life (AQoL) measure. Aust N Z J Public Health.

[50] Cuthbertson BH, Elders A, Hall S, Taylor J, Maclennan G, Mackirdy F, Mackenzie SJ, the Scottish Critical Care Trials G, the Scottish Intensive Care Society Audit G (2013). Mortality and quality of life in the five years after severe sepsis. Crit Care.

[51] Berney SC, Harrold M, Webb SA, Seppelt I, Patman S, Thomas PJ, Denehy L (2013). Intensive care unit mobility practices in Australia and New Zealand: a point prevalence study. Crit Care Resusc.

[52] Denehy L, Nordon-Craft A, Edbrooke L, Malone D, Berney S, Schenkman M, Moss M (2014). Outcome measures report different aspects of patient function three months following critical care. Intensive Care Med.

[53] Puthucheary ZA, Denehy L (2015). Exercise interventions in critical illness survivors: understanding inclusion and stratification criteria. Am J Respir Crit Care Med.

[54] Orwelius L, Nordlund A, Edell-Gustafsson U, Simonsson E, Nordlund P, Kristenson M, Bendtsen P, Sjoberg F (2005). Role of preexisting disease in patients' perceptions of health-related quality of life after intensive care. Crit Care Med.

[55] Orwelius L, Nordlund A, Nordlund P, Simonsson E, Backman C, Samuelsson A, Sjoberg F (2010). Pre-existing disease: the most important factor for health related quality of life long-term after critical illness: a prospective, longitudinal, multicentre trial. Crit Care.

[56] Iwashyna TJ (2012). Trajectories of recovery and dysfunction after acute illness, with implications for clinical trial design. Am J Respir Crit Care Med.

[57] Cuthbertson BH, Wunsch H (2016). Long-term outcomes after critical illness. The best predictor of the future is the past. Am J Respir Crit Care Med.

[58] Herridge MS, Batt J, Santos CD (2014). ICU-acquired weakness, morbidity, and death. Am J Respir Crit Care Med.

[59] Herridge MS, Chu LM, Matte A, Tomlinson G, Chan L, Thomas C, Friedrich JO, Mehta S, Lamontagne F, Levasseur M, Ferguson ND, Adhikari NK, Rudkowski JC, Meggison H, Skrobik Y, Flannery J, Bayley M, Batt J, Santos CD, Abbey SE, Tan A, Lo V, Mathur S, Parotto M, Morris D, Flockhart L, Fan E, Lee CM, Wilcox ME, Ayas N, Choong K, Fowler R, Scales DC, Sinuff T, Cuthbertson BH, Rose L, Robles P, Burns S, Cypel M, Singer L, Chaparro C, Chow CW, Keshavjee S, Brochard L, Hebert P, Slutsky AS, Marshall JC, Cook D, Cameron JI, Investigators RP, Canadian Critical Care Trials G (2016). The RECOVER program: disability risk groups and 1-year outcome after 7 or more days of mechanical ventilation. Am J Respir Crit Care Med.

[60] Soliman IW, de Lange DW, Peelen LM, Cremer OL, Slooter AJ, Pasma W, Kesecioglu J, van Dijk D (2015). Single-center large-cohort study into quality of life in Dutch intensive care unit subgroups, 1 year after admission, using EuroQoL EQ-6D-3L. J Crit Care.

[61] Orwelius L, Fredrikson M, Kristenson M, Walther S, Sjoberg F (2013). Health-related quality of life scores after intensive care are almost equal to those of the normal population: a multicenter observational study. Crit Care.

[62] Maley J, Brewster I, Mayoral I, Siruckova R, Adams S, McGraw K, Piech A, Detsky M, Mikkelsen M (2016). Resilience in survivors of critical illness in the context of the survivors' experience and recovery. Ann Am Thorac Soc.

[63] Mikkelsen ME, Jackson JC, Hopkins RO, Thompson C, Andrews A, Netzer G, Bates DM, Bunnell AE, Christie LM, Greenberg SB, Lamas DJ, Sevin CM, Weinhouse G, Iwashyna TJ (2016). Peer support as a novel strategy to mitigate post-intensive care syndrome. AACN Adv Crit Care.

[64] Mikkelsen ME, Christie JD, Lanken PN, Biester RC, Thompson BT, Bellamy SL, Localio AR, Demissie E, Hopkins RO, Angus DC (2012). The adult respiratory distress syndrome cognitive outcomes study: long-term neuropsychological function in survivors of acute lung injury. Am J Respir Crit Care Med.

[65] Bienvenu OJ, Williams JB, Yang A, Hopkins RO, Needham DM (2013). Posttraumatic stress disorder in survivors of acute lung injury: evaluating the impact of event scale-revised. Chest.

[66] Cameron JI, Chu LM, Matte A, Tomlinson G, Chan L, Thomas C, Friedrich JO, Mehta S, Lamontagne F, Levasseur M, Ferguson ND, Adhikari NK, Rudkowski JC, Meggison H, Skrobik Y, Flannery J, Bayley M, Batt J, dos Santos C, Abbey SE, Tan A, Lo V, Mathur S, Parotto M, Morris D, Flockhart L, Fan E, Lee CM, Wilcox ME, Ayas N, Choong K, Fowler R, Scales DC, Sinuff T, Cuthbertson BH, Rose L, Robles P, Burns S, Cypel M, Singer L, Chaparro C, Chow CW, Keshavjee S, Brochard L, Hebert P, Slutsky AS, Marshall JC, Cook D, Herridge MS, Investigators RP, Canadian Critical Care Trials G (2016). One-year outcomes in caregivers of critically ill patients. N Engl J Med.

[67] Haines KJ, Denehy L, Skinner EH, Warrillow S, Berney S (2015). Psychosocial outcomes in informal caregivers of the critically ill: a systematic review. Crit Care Med.

