# Supplementary material for: Long-term recovery following critical illness in an Australian cohort
Source: J Intensive Care. 2018 Feb 5;6:8. doi: 10.1186/s40560-018-0276-x (PMC5800039; doi:10.1186/s40560-018-0276-x)
Supplement: Additional file 1: — Appendix E1. Independent living status and employment status questions. Appendix E2. Cause of death information provided by the Victorian Births and Deaths Registry. (DOC 44 kb) [file 40560_2018_276_MOESM1_ESM.doc]

**Online Data Supplement**

Long-term Recovery Following Critical Illness in an Australian Cohort

Authors:

Dr. Kimberley J. Haines

Associate Professor Sue Berney

Dr. Stephen Warrillow

Professor Linda Denehy

***Appendix E1 – Independent living status and employment status questions***

Independent living status:

Classified according to the following categories:

1. Home and independent with usual daily activities
2. Home requiring assistance with usual daily activities
3. Supportive accommodation (e.g. hostel, not own home)
4. Nursing home (e.g. requiring full nursing assistance with activities of daily living)

Return to work:

Were you working prior to your ICU admission?

- If so, how many hours per week?
- If not, why not? Have you returned to work after your ICU admission?
- If so, after how many months following ICU discharge?
- If so, is this the same work you did before?
- If not, what are the reasons for the change in work?
- How many hours per week do you work currently?
- If unable to return to work following ICU discharge please explain why?

***Appendix E***2 – Cause of death information provided by the Victorian Births and Deaths Registry

| **Primary cause of death** | **n** |
| --- | --- |
| ***Cancer*** |  |
| Metastatic breast cancer | 1 |
| Cancer of bladder/prostate | 2 |
| Oesophageal adenocarcinoma | 1 |
| Squamous cell carcinoma of the tongue | 2 |
|  |  |
| ***Respiratory*** |  |
| Pneumonia on background of chronic respiratory lung disease | 6 |
| Pneumonia with ARDS | 2 |
| Pneumonia | 6 |
|  |  |
| ***Cardiac*** |  |
| Ischaemic heart disease | 5 |
| Cardiac shock/ failure | 8 |
|  |  |
| ***Sepsis*** |  |
| Severe sepsis | 7 |
|  |  |
| ***Hepatic*** |  |
| End stage liver disease | 2 |
|  |  |
| ***Neurological*** |  |
| Ischaemic stroke | 2 |
|  |  |
| ***Other*** |  |
| Drug toxicity | 1 |

*Not all cause of death information could be obtained for all patients deceased from the state registry due to data/information not provided*
